# Diverse Empirical Evidence on Epidemiological Transition in Low- and Middle-Income Countries: Population-Based Findings from INDEPTH Network Data

**DOI:** 10.1371/journal.pone.0155753

**Published:** 2016-05-17

**Authors:** Ailiana Santosa, Peter Byass

**Affiliations:** 1 Umeå Centre for Global Health Research, Unit of Epidemiology and Global Health, Department of Public Health and Clinical Medicine, Umeå University, 90187 Umeå, Sweden; 2 Center for Demographic and Ageing Research, Umeå University, 90187 Umeå, Sweden; 3 MRC/Wits Rural Public Health and Health Transitions Research Unit, School of Public Health, Faculty of Health Sciences, University of the Witwatersrand, Johannesburg, South Africa; UNAIDS, TRINIDAD AND TOBAGO

## Abstract

**Background:**

Low- and middle-income countries are often described as being at intermediate stages of epidemiological transition, but there is little population-based data with reliable cause of death assignment to examine the situation in more detail. Non-communicable diseases are widely seen as a coming threat to population health, alongside receding burdens of infection. The INDEPTH Network has collected empirical population data in a number of health and demographic surveillance sites in low- and middle-income countries which permit more detailed examination of mortality trends over time.

**Objective:**

To examine cause-specific mortality trends across all ages at INDEPTH Network sites in Africa and Asia during the period 1992–2012. Emphasis is given to the 15–64 year age group, which is the main focus of concern around the impact of the HIV pandemic and emerging non-communicable disease threats.

**Methods:**

INDEPTH Network public domain data from 12 sites that each reported at least five years of cause-specific mortality data were used. Causes of death were attributed using standardised WHO verbal autopsy methods, and mortality rates were standardised for comparison using the INDEPTH standard population. Annual changes in mortality rates were calculated for each site.

**Results:**

A total of 96,255 deaths were observed during 9,487,418 person years at the 12 sites. Verbal autopsies were completed for 86,039 deaths (89.4%). There were substantial variations in mortality rates between sites and over time. HIV-related mortality played a major part at sites in eastern and southern Africa. Deaths in the age group 15–64 years accounted for 43% of overall mortality. Trends in mortality were generally downwards, in some cases quite rapidly so. The Bangladeshi sites reflected populations at later stages of transition than in Africa, and were largely free of the effects of HIV/AIDS.

**Conclusions:**

To some extent the patterns of epidemiological transition observed followed theoretical expectations, despite the impact of the HIV pandemic having a major effect in some locations. Trends towards lower overall mortality, driven by decreasing infections, were the general pattern. Low- and middle-income country populations appear to be in an era of rapid transition.

## Introduction

All populations follow particular epidemiological transition trajectories, but for many low- and middle-income countries the process is often unclear because of a lack of reliable longitudinal data, particularly on causes of death. Poorer countries are less likely to have reliable data for characterising long-term transitions [1]. The World Health Organization estimated that in 2003 only 64/192 member states had essentially complete death registration [2], seriously prejudicing long-term consideration of transitional processes even in contexts where more recent progress with civil registration has been made. Many studies have described changes in population age-sex structures and cause-specific mortality patterns, often quoting the concept of epidemiological transition theory [3], but sometimes lacking adequate data. Epidemiological transition theory has been used as a model to describe or justify shifts over time in some contexts, generally from communicable diseases towards non-communicable diseases [4]. The Global Burden of Disease project estimated that 16 out of 38 million deaths in 2010 occurred in low- and middle-income countries, with 82% of those deaths occurring before the age of 60 [5]. Premature adult mortality, defined by the World Health Organization as before 70 years of age in the context of non-communicable disease control [6], is therefore a major problem for low- and middle-income countries. Many low- and middle-income countries currently have significant dual burdens of communicable and non-communicable diseases, with the HIV/AIDS pandemic adding complexity to the process of epidemiological transition [7].

Given the scarcity of national longitudinal cause-specific mortality data from low- and middle-income countries, an alternative source of empirical data comes from health and demographic surveillance sites operated under the umbrella of the INDEPTH Network [8]. Although the national representativity of these sites is not easy to establish, the INDEPTH data provide high quality and methodologically consistent longitudinal health and demographic data within defined local populations which can help to understand how mortality transitions progress over time. The aim of this paper, using cause-specific mortality data from INDEPTH sites offering at least five years of continuous observations, is to compare on an empirical basis within-site mortality trends over time and between-site mortality differences at the same points in time. Specific attention is given to the 15–64 year age group, among whom mortality has been particularly heavily impacted by the HIV pandemic in many locations, as well as being the focus of emerging concerns about rising levels of non-communicable diseases and related premature mortality.

## Methods

**Fig 1:**
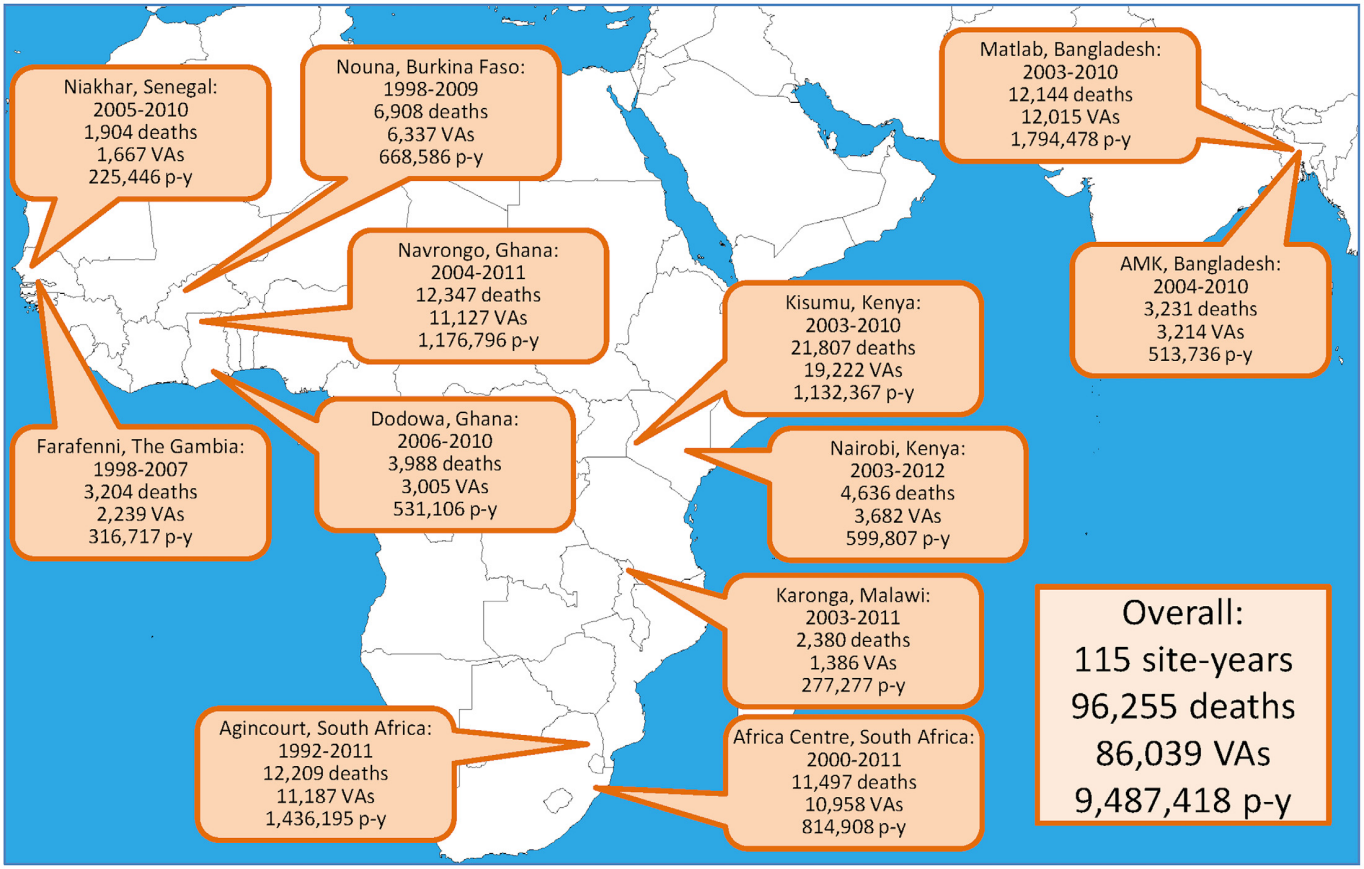
Map showing 12 INDEPTH Network sites with time period of observation, deaths, verbal autopsies and person-time observed.

Data were sourced from the INDEPTH cause-specific mortality public-domain dataset [9], based on primary data collected by constituent health and demographic surveillance sites. Twelve sites contributed data over five or more years, as shown in Fig 1, amounting to 115 site-years. In all, 96,255 deaths were documented, for which 86,039 (89.4%) successfully completed a verbal autopsy interview. These deaths occurred against 9,487,418 person-years of observation.

The detailed methodology behind this dataset is described in detail elsewhere [10] and the methods and sources are summarised in Box 1. All of the 12 sites involved covered entire populations, and, apart from the urban Nairobi, Kenya, site, all were located in rural or semi-rural areas. The Karonga, Malawi, site did not contribute verbal autopsy and cause of death data for children aged under 12 years, though the childhood deaths are included in the overall figures. The InterVA-4 model [11, 12] requires background information on local levels of malaria and HIV/AIDS mortality, dichotomised as high (greater than around 1% of all deaths) or low. In the 12 included sites, the background level for malaria was high in the West African sites plus Kisumu, Kenya and Karonga, Malawi; HIV was high for the sites in Kenya, Malawi and South Africa. Further details of the individual sites contributing data are available as separate site papers [13–24].

**Box 1. Summary of methodology (source: (11))**

**Age-sex-time standardisation**

To avoid effects of differences and changes in age-sex structures of populations, mortality fractions and rates have been adjusted using the INDEPTH 2013 population standard (12). A weighting factor was calculated for each site, age group, sex and year category in relation to the standard for the corresponding age group and sex, and incorporated into the overall dataset. This is referred to in this paper as age-sex-time standardisation in the contexts where it is used.

**Cause of death assignment**

The InterVA-4 (version 4.02) probabilistic model was used for all the cause of death assignments in the overall dataset (13). InterVA-4 is fully compliant with the WHO 2012 Verbal Autopsy standard and generates causes of death categorised by ICD-10 groups (14). The data reported here were collected before the WHO 2012 VA standard was available, but were transformed into the WHO 2012 and InterVA-4 format to optimise cross-site standardisation in cause of death attribution. For a small proportion of deaths VA interviews were not successfully completed; a few others contained inadequate information to arrive at a cause of death. InterVA-4 assigns causes of death (maximum 3) with associated likelihoods; thus cases for which likely causes did not total 100% were also assigned a residual indeterminate component. This served as a means of encapsulating uncertainty in cause of death at the individual level within the overall dataset, as well as accounting for 100% of every death.

**Overall dataset**

The overall public-domain dataset (9) thus contains between one and four records for each death, with the sum of likelihoods for each individual being unity. Each record includes a specific cause of death, its likelihood and its age-sex-time weighting.

Age-sex-year standardisation using the INDEPTH standard population [25] was incorporated into the public-domain dataset, and was used here in order to compare age-standardised death rates (ASDR) over time and between sites while controlling for variations in the age-sex structure of the site populations over time. Thus observed variations in mortality are not consequences of differences and changes in population structures. For assessing varying degrees of epidemiological transition, in the absence of any standard metric we compared non-communicable causes of death with all other non-indeterminate causes in the 15–64 year age group. Annual changes in standardised mortality rates were calculated using linear regression.

The INDEPTH public-domain dataset was derived from primary data collected separately by each participating site. In all cases the primary data collection was covered by site-level ethical approvals relating to on-going demographic surveillance in those specific locations. No individual identity or household location data were included in the secondary data and no specific ethical approvals were required for these secondary analyses.

## Results

Within the overall 96,255 deaths and 9,487,418 person-years observed, verbal autopsies were completed for 86,039 deaths. Thus there were 10,216 deaths (10.6%) with no VA data, and in addition InterVA-4 was unable to assign any definite cause(s) of death (i.e. arrived at a 100% indeterminate outcome) in 3,981 (4.1%) of cases. As described in Box 1, residual fractions assigned as indeterminate amounted to a further 6,553 (6.8%) of cases. There were major variations in mortality between sites and over time. There was a three-fold variation in standardised mortality rates between sites (Kisumu, Kenya being the highest and Matlab, Bangladesh being the lowest) during the periods of observation. The within-site rates of change in mortality also varied widely. Most sites showed a decrease in mortality rates over time, but some showed an increase, such as the Agincourt, South Africa, site, which recorded an overall increase in mortality because its period of observation started in 1992, before the major mortality impact of the HIV pandemic took effect.

**Fig 2:**
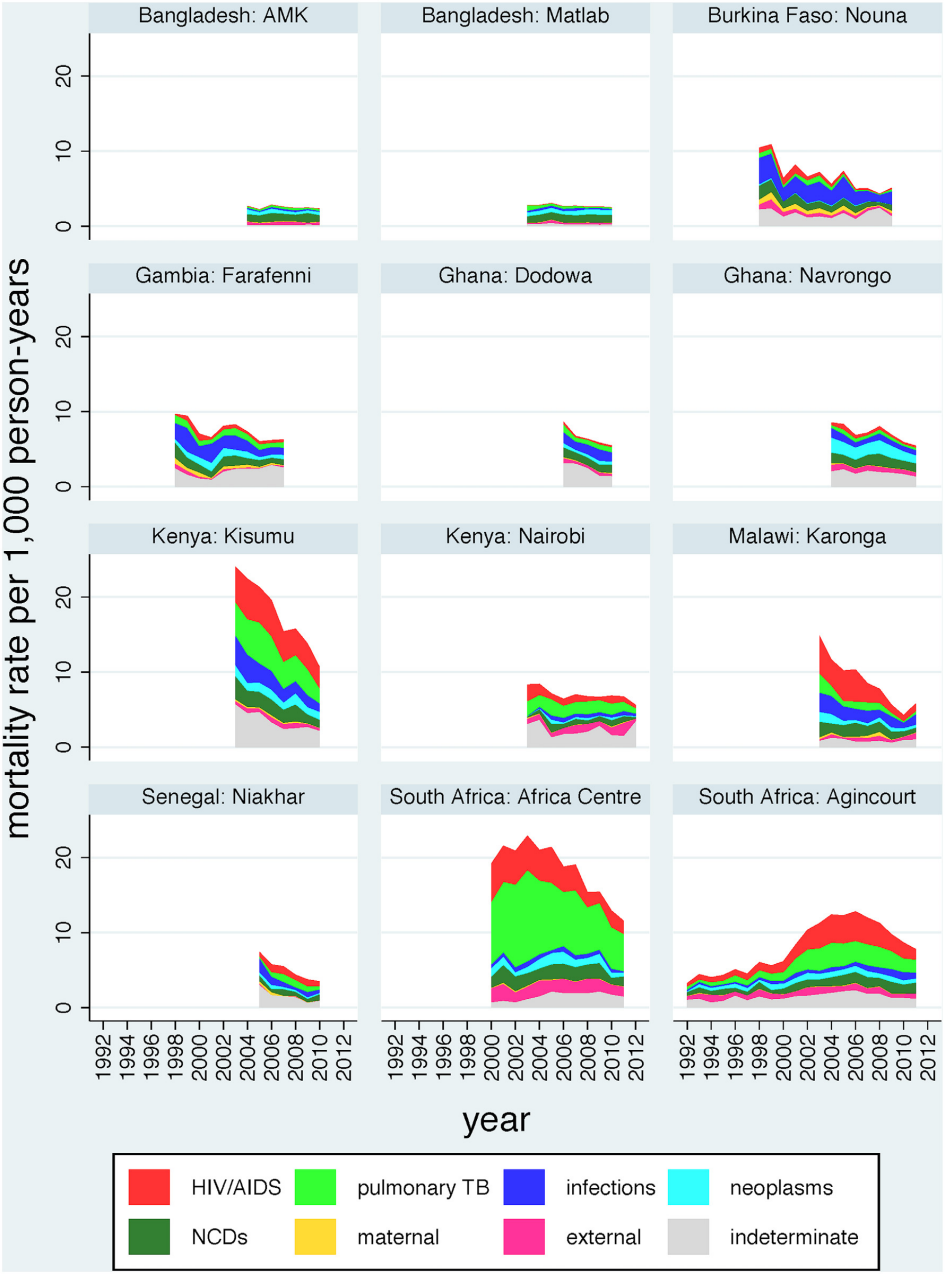
Trends in age-sex-time standardised death rates (ASDR) per 1,000 person-years for all causes of death (by verbal autopsy) among adults aged 15 to 64 years in 12 INDEPTH Network population sites during 1992 to 2012, for 41,666 deaths in 5,303,232 person-years observed.

Specifically for the younger adult (15 to 64 year) age group, there were 41,666 deaths in 5,303,232 person-years observed, as shown in Table 1. In this age group, there was a seven-fold variation in ASDR between sites (Africa Centre, South Africa being the highest and AMK, Bangladesh, being the lowest) during the periods of observation. Fig 2 shows the changes in ASDR for the 15–64 year age group over time at each site, split into major causes of death (HIV/AIDS, pulmonary tuberculosis, other infections, neoplasms, non-communicable diseases, maternal, external and indeterminate causes). Within-site rates of change also varied considerably in the 15–64 year age group, from a fall of 15.3% per year in Niakhar, Senegal, to 1.5% per year in Matlab, Bangladesh; the Agincourt, South Africa site showed an overall increase of 5.2% per year because of the dynamics of HIV-related mortality.

**Table 1:** Age-sex-time standardised death rates (ASDR) per 1,000 person-years for all deaths in the 15–64 years age group in twelve INDEPTH Network sites, 41,666 deaths in 5,303,232 person-years observed.

Site	Period	Person-years	Deaths	ASDR	% annual change (95% CI)
Bangladesh: AMK	2004–2010	327,313	1,001	2.5	-1.5 (-0.8, -2.3)
Bangladesh: Matlab	2003–2010	1,076,019	3,450	2.7	-1.8 (-1.4, -2.2)
Burkina Faso: Nouna	1998–2009	323,618	1,787	6.2	-8.0 (-6.7, -9.3)
Gambia: Farafenni	1998–2007	162,231	1,077	7.2	-4.7 (-3.4, -6.0)
Ghana: Dodowa	2006–2010	292,679	1,835	6.5	-11.1 (-10, -12.5)
Ghana: Navrongo	2004–2011	662,957	5,200	7.1	-5.4 (-4.8, -6.0)
Kenya: Kisumu	2003–2010	556,796	8,928	17.7	-10.6 (-10, -11.2)
Kenya: Nairobi	2003–2012	408,614	2,775	7.0	-3.2 (-2.6, -3.9)
Malawi: Karonga	2003–2010	132,282	907	8.3	-14.1 (-11.8, -16.4)
Senegal: Niakhar	2005–2010	113,821	516	5.0	-15.3 (-12.2, -18.4)
South Africa: Africa Centre	2000–2011	428,951	7,187	18.3	-4.7 (-4.2, -5.2)
South Africa: Agincourt	1992–2011	817,951	7,003	8.4	5.2 (4.7, 5.7)

**Fig 3:**
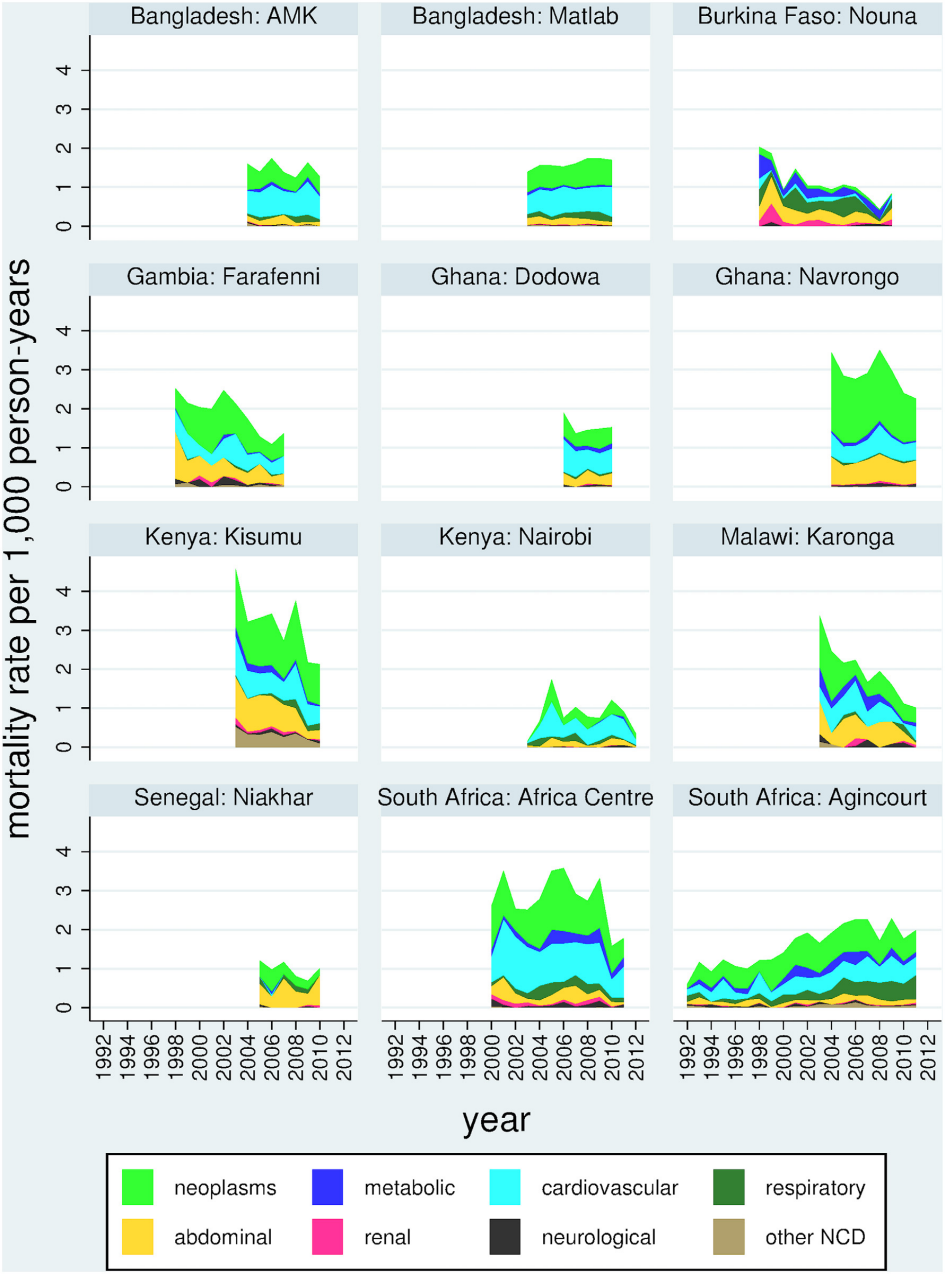
Trends in age-sex-time standardised death rates (ASDR) per 1,000 person-years for non-communicable diseases (by verbal autopsy) among adults aged 15 to 64 years in 12 INDEPTH Network population sites during 1992 to 2012, for 10,172 deaths in 5,303,232 person-years observed.

**Fig 4:**
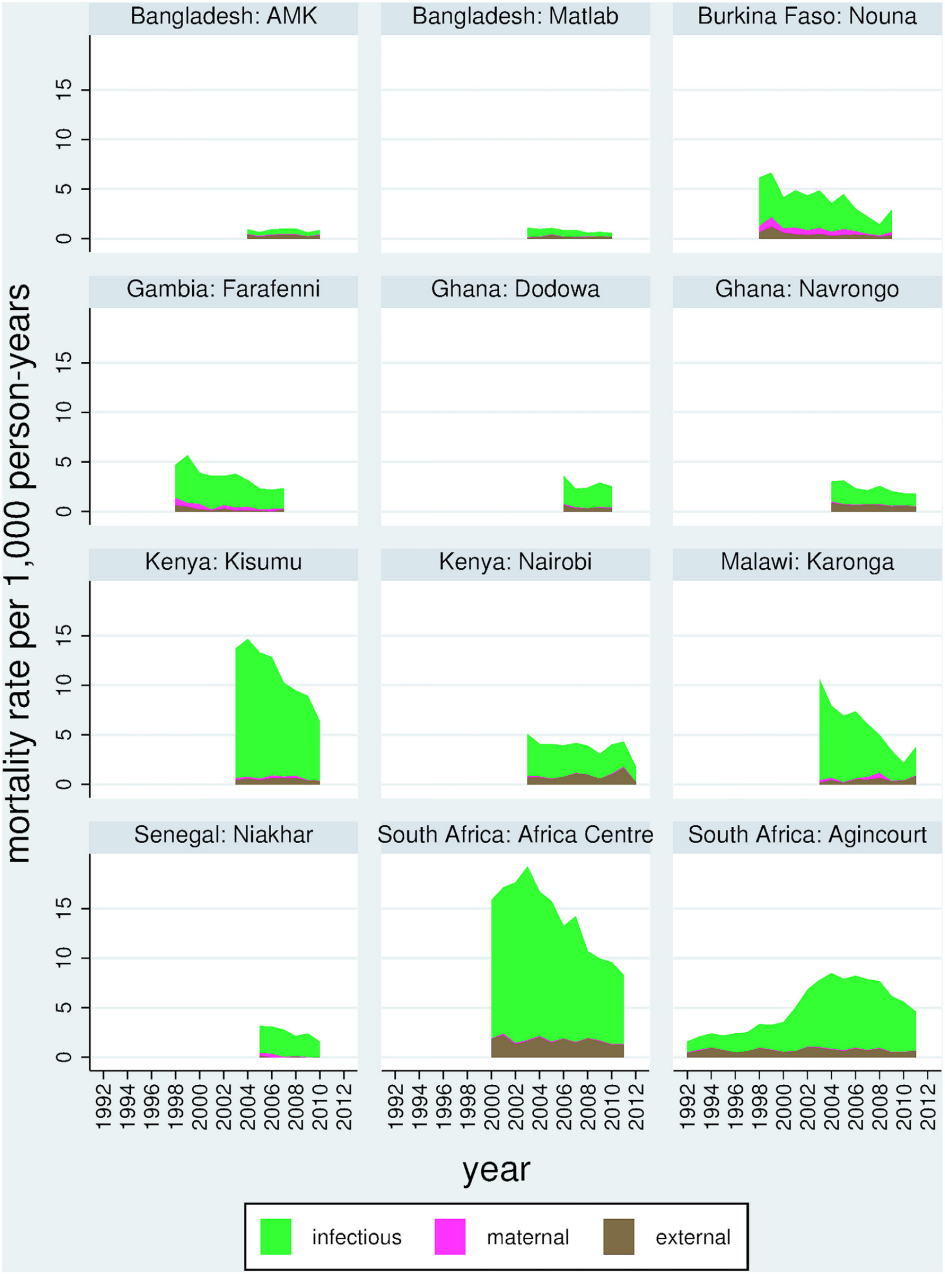
Trends in age-sex-time standardised death rates (ASDR) per 1,000 person-years for infectious, maternal and external causes of death (by verbal autopsy) among adults aged 15 to 64 years in 12 INDEPTH Network population sites during 1992–2012, for 23,432 deaths in 5,303,232 person-years observed.

In the 15–64 year age group, 10,172/41,666 deaths (24.4%) were due to non-communicable diseases. Non-communicable disease mortality was taken as the WHO 2012 verbal autopsy standard cause of death chapters that relate to non-communicable diseases [12], namely neoplasms, metabolic diseases, cardiovascular diseases, chronic respiratory diseases, abdominal diseases (including liver disease), renal diseases, neurological diseases and other non-communicable diseases. For purposes of comparison, the remaining VA cause of death chapters, namely infectious, maternal and external causes, but excluding indeterminate categories [12], were used, accounting for 23,432/41,666 deaths (56.2%). Table 2 shows ASDRs for both of these major mortality categories by site. ASDR for non-communicable causes varied from 0.8 per 1,000 person-years in Nairobi, Kenya to 3.1 in Kisumu, Kenya. Annual rates of change varied widely from a decrease of 13.5% in Karonga, Malawi to an increase of 4.5% in Agincourt, South Africa. For the infectious, maternal and external category, ASDR varied from 0.8 per 1,000 person-years in Matlab, Bangladesh to 13.9 per 1,000 person-years in the Africa Centre, South Africa. Annual rates of change varied from a decrease of 16.3% in Karonga, Malawi to an increase of 6.1% in Agincourt, South Africa. Ratios of ASDR for non-NCD to NCD causes were much lower in Bangladesh (lowest 0.5 in Matlab, Bangladesh) than in African sites (highest 5.0 in the Africa Centre, South Africa). In almost all sites, ASDR for infectious, maternal and external causes declined more rapidly than the ASDR for non-communicable causes; nevertheless in all but one of the African sites ASDR for infectious, maternal and external causes exceeded ASDR for non-communicable causes. Fig 3 shows trends in non-communicable disease mortality, and Fig 4 similarly shows infectious, maternal and external causes, by site and over time for the WHO VA cause of death chapters.

**Table 2:** Age-sex-time standardised death rates (ASDR) per 1,000 person-years for 41,666 deaths in 5,303,232 person-years among the 15–64 year age group in twelve INDEPTH Network sites.

Site	Period	Person-years	Non-communicable causes			Infectious, maternal and external			ASDR ratio non-NCD:NCD	Indeterminate or no VA done	
			Deaths	ASDR	% annual change (95% CI)	Deaths	ASDR	% annual change (95% CI)		Deaths	ASDR
Bangladesh: AMK	2004–2010	327,313	587	1.5	-2.4 (-1.2, -3.6)	333	0.9	-0.6 (-0.2, -1.4)	0.6	81	0.2
Bangladesh: Matlab	2003–2010	1,076,019	2,060	1.6	2.7 (2.0, 3.4)	1,022	0.8	-9.3 (-7.5, -11.1)	0.5	368	0.3
Burkina Faso: Nouna	1998–2009	323,618	280	1.0	-10.5 (-6.9, -14.1)	1,039	3.6	-10.3 (-8.5, -12.1)	3.6	468	1.6
Gambia: Farafenni	1998–2007	162,231	254	1.7	-7.9 (-4.6, -11.2)	478	3.2	-10.4 (-7.7, -13.1)	1.9	344	2.3
Ghana: Dodowa	2006–2010	292,679	419	1.5	-4.0 (-2.1, -5.9)	775	2.7	-5.5 (-3.9, -7.1)	1.8	641	2.3
Ghana: Navrongo	2004–2011	662,957	2,129	2.9	-3.8(-3.0, -4.6)	1,667	2.3	-7.8 (-6.5, -9.1)	0.8	1,404	1.9
Kenya: Kisumu	2003–2010	556,796	1,581	3.1	-8.2 (-6.9, -9.6)	5,594	11.1	-10.1 (-9.3, -10.9)	3.6	1,752	3.5
Kenya: Nairobi	2003–2012	408,614	295	0.8	0.4 (0.3, 1.1)	1,584	3.8	-4.9 (-3.8, -6.0)	4.8	895	2.3
Malawi: Karonga	2003–2010	132,282	194	1.8	-13.5 (-8.7, -18.3)	613	5.5	-16.3 (-13.4, -19.2)	3.0	100	0.9
Senegal: Niakhar	2005–2010	113,821	100	1.0	-6.5 (-1.7, -11.3)	258	2.5	-12.3 (-8.3, -16.3)	2.5	158	1.5
South Africa: Africa Centre	2000–2011	428,951	1,010	2.8	-2.4 (-1.5, -3.3)	5,575	13.9	-6.5 (-5.9, -7.2)	5.0	602	1.6
South Africa: Agincourt	1992–2011	817,951	1,262	1.7	4.5 (3.4, 5.6)	4,494	5.3	6.1 (5.4, 6.8)	3.2	1,246	1.5

**Fig 5:**
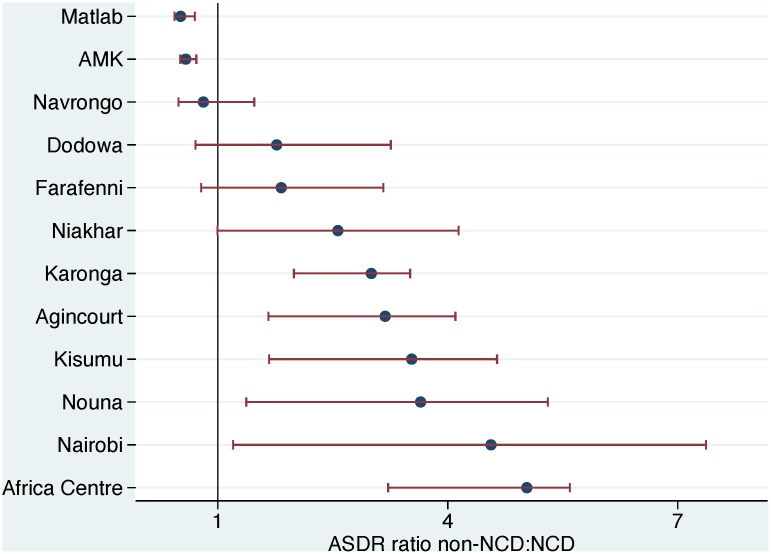
Sensitivity analysis for age-sex-time standardised death rate (ASDR) ratios for non-NCD: NCD causes (as shown in Table 2), among adults aged 15 to 64 years in 12 INDEPTH Network population sites during 1992 to 2012, for 41,666 deaths in 5,303,232 person-years observed. Bars show ranges representing worst-case error scenarios where all indeterminate causes of death were assigned either to the non-NCD or NCD group.

To address concerns about the possible effects of the indeterminate cause group on the ratios of non-NCD to NCD mortality, we undertook a sensitivity analysis in which, as worst-case scenarios, all the indeterminate cases were added into either the non-NCD or NCD groups. The effects of this on the ASDR non-NCD: NCD ratios for each site are shown in Fig 5.

## Discussion

This study, based on highly standardised methods across 12 INDEPTH Network sites, showed considerable differences in mortality patterns between sites and over time, reflecting epidemiological transition processes driven by several factors. It provides important insights into mortality transitions from contexts where requisite longitudinal population-based cause of death data are not readily available. The sites represented a wide spectrum of HIV/AIDS epidemiology, as previously reported in detail [26], allowing exploration of the effects of the HIV pandemic on epidemiological transition, in addition to Omran’s basic ideas of populations progressing from traditional infectious to non-communicable causes of death [3].

### Overall changes in mortality patterns

Broadly speaking, these site populations all showed positive signs in terms of moving towards lower mortality, with reducing rates of infectious causes. One apparent exception was the Agincourt, South Africa, site, but this largely arose because the site started its data series at an earlier point, before the impact of the HIV pandemic was strongly apparent. In more recent years overall mortality in Agincourt declined as HIV/AIDS mortality reduced with anti-retroviral treatment [27]. One interesting feature of the mortality changes observed here is the generally rapid rates at which changes have occurred in the early 21^st^ century. Omran’s original consideration of population trends, leading to the postulation of his theory, was predicated on changes that occurred over decades or even centuries. Clearly current transitions in low- and middle-income countries are proceeding much more rapidly than they did earlier in the now high-income countries.

The sites represented here fall into three main groups. Firstly, the two Bangladeshi sites show very similar patterns of low mortality, against a background of increasing life expectancy, with relatively little infectious cause mortality of any kind, and almost no HIV/AIDS [13, 14]. External causes of death, including in particular drowning in the very watery environment, are a continuing concern [28]. Secondly, the sites in West Africa form a relatively coherent group with high burdens of infectious disease but quite low HIV/AIDS-related mortality. A large part of the infectious burden was due to malaria, as previously reported in detail [29]. Thirdly, the sites in eastern and southern Africa are characterised by higher burdens of HIV/AIDS-related mortality, with varying degrees of other infections. The Kisumu, Kenya and Karonga, Malawi, sites have an appreciable malaria burden in addition to HIV/AIDS, whereas the sites in South Africa have very low malaria exposure [29]. The Nairobi, Kenya, site is something of a special case compared with other sites, since it is located in the urban slums, and many residents have moved there seeking economic opportunities, reflecting a degree of self-selection. In addition, the altitude of Nairobi city makes it more or less malaria-free. These results build on previous findings from the INDEPTH Network, compiled before standardised methods for processing verbal autopsy data were in widespread use [30].

### Implications of the HIV pandemic for epidemiological transition

When the original principles of epidemiological transition were developed, the prospect of a new global infectious pandemic that could become a leading cause of death in some contexts was not given very much consideration. There is clear evidence in this study of the major disruption to epidemiological transition arising from the HIV pandemic, both in terms of mortality directly attributed as HIV/AIDS-related, and also elevated rates of other HIV-associated causes. The younger part of the adult age spectrum, taken here as 15–64 years for consistency with the WHO 2012 verbal autopsy categories [31], is a critical group when considering epidemiological transition. This age group classically benefits from averted infectious deaths and rapidly accumulates increased life expectancy. However, the HIV pandemic particularly hit this sexually active age group in the pre-treatment era, with massive consequences for mortality in the worst affected populations. This was clearly evident in the sites from eastern and southern Africa. However, the effects of being HIV-positive on other causes of death also have to be taken into account. A study from the ALPHA Network showed substantially raised risks of death from many causes among people living with HIV [32], and previous INDEPTH analyses showed associations between rates of HIV/AIDS mortality and non-communicable disease mortality within the same populations [33]. Going forward, there are considerable uncertainties about likely cause of death patterns among people on antiretroviral therapy, particularly after some decades [34].

### Contemporary global comparisons

Since there is no single metric for epidemiological transition, it is not possible to locate specific population findings on a global scale. Nevertheless, in terms of Omran’s stages of transition [3], these twelve populations seem to be in the realm of “receding pandemics”, including now the HIV pandemic. The World Health Organization 25x25 target (a 25% reduction in premature non-communicable disease mortality from 2010 to 2025) was set for all Member States irrespective of their stage of epidemiological transition in 2010 [6], corresponding to an annual reduction of approximately 1.5%. Most of the sites included here appear to have been on a steeper downward trend in non-communicable disease mortality at the start of the target period. As yet there is little empirical evidence on progress towards this target, but Sweden, already at a late stage of epidemiological transition, achieved a 25% reduction in premature non-communicable disease mortality during an earlier equivalent period 1991–2006. Three-quarters of deaths among Swedes in the 40–70 year age range were due to non-communicable disease, an appreciably higher proportion than in any of the sites here, reflecting Sweden’s later stage of transition [35].

### Strengths and limitations of the study

A major advantage of the INDEPTH mortality data is that all the records relate to known individuals within defined populations, precluding the need for modelled estimation methods. All of the populations are under active surveillance to register deaths, achieving good coverage [8]. Furthermore, standardised verbal autopsy methods based on the WHO 2012 standard [31] were applied across all sites, so there was no scope for various local doctors to individually come to systematically different conclusions about causes of death. Nevertheless, the locations and settings of the participating INDEPTH Network sites are quite serendipitous, so that the ranges of findings across all the sites, rather than any aggregated measures, have to be the endpoint. Even though the INDEPTH dataset used here is the largest of its kind available for low- and middle-income countries, longer periods of observation and larger population coverage would have been desirable. Ideally it would be better to have all the sites reporting for the same time period; if all the sites had been documenting cause of death for as long as the Agincourt, South Africa, site, the dataset would be correspondingly richer. Nevertheless, the scale of the database is impressive; the 115 site-years of verbal autopsy data reported here can be compared with a total of around 500 site-years of verbal autopsy data in the entire Global Burden of Disease database [36].

Any cause of death assignment process on a whole-population basis results in a proportion of cases with no determinate cause, as well as degree of uncertainty in causes which are assigned. Physician certification of death normally ignores the individual-level uncertainty associated with cause assignment, whereas the use of a probabilistic model such as InterVA-4 facilitates quantifying individual uncertainty. When deaths occurring in a population are followed up later with VA, inevitably in a proportion follow-up fails, or respondents provide insufficient detail to arrive at a cause. Thus the proportion of cases assigned as indeterminate in the INDEPTH dataset is not surprising; but the methodological issue of how those deaths, if we knew their “true” causes, might affect results is important. In these analyses we have presented the worst-case scenarios in which either all the indeterminate cases were truly NCD cases, or truly non-NCD cases (Fig 5). Even making these extreme and very unlikely assumptions, there are still considerable differences between the sites in terms of the ratios of non-NCD to NCD mortality. In reality, the unknown “true” causes of death for the indeterminate cases are likely to be much more evenly balanced between the two groups, and thus affect findings to a lesser extent.

## Conclusions

The trends in various causes of death presented in this study generally confirm that epidemiological transitions in the low- and middle-income countries represented here are progressing in some ways in accordance with the expectations of Omran’s theory [3], though heavily perturbed in many settings by the HIV pandemic. The processes of transition towards lower mortality, and towards non-communicable rather than infectious causes of death, are in many cases proceeding rapidly, and much faster than was the case in countries now at a late stage of transition. Understanding these transitional processes provides an important source of information for population health and development.
